# Sliding mode tracking control of a class of fractional-order nonstrict-feedback nonlinear systems

**DOI:** 10.1007/s11071-024-09789-0

**Published:** 2024-07-05

**Authors:** Reza Mohsenipour, Daniel Massicotte

**Affiliations:** 1 Department of Electrical and Computer Engineering, University of Quebec at Trois-Rivieres, Trois-Rivières, QC Canada; 2 Department of Engineering, University of Cambridge, Cambridge, CB2 1PZ UK

**Keywords:** Sliding mode control, Nonlinear system, Nonstrict-feedback, Fractional-order, Uncertainty

## Abstract

Since the Leibniz rule for integer-order derivatives of the product of functions, which includes a finite number of terms, is not true for fractional-order (FO) derivatives of that, all sliding mode control (SMC) methods introduced in the literature involved a very limited class of FO nonlinear systems. This article presents a solution for the unsolved problem of SMC of a class of FO nonstrict-feedback nonlinear systems with uncertainties. Using the Leibniz rule for the FO derivative of the product of two functions, which includes an infinite number of terms, it is shown that only one of these terms is needed to design a SMC law. Using this point, an algorithm is given to design the controller for reference tracking, that significantly reduces the number of design parameters, compared to the literature. Then, it is proved that the algorithm has a closed-form solution which presents a straightforward tool to the designer to obtain the controller. The solution is applicable to the systems with a mixture of integer-order and FO dynamics. Stability and finite-time convergence of the offered control law are also demonstrated. In the end, the availability of the suggested SMC is illustrated through a numerical example arising from a real system.

## Introduction

Sliding mode control (SMC) is considered as one of the most popular, applicable methodologies among robust control design methods to deal with nonlinear systems suffering from uncertainties and disturbances [6]. On the other hand, with advances in FO calculus, many real-world systems have been modeled or controlled with FO differential equations to reach a better performance, compared to integer-order differential equations [17, 25, 35]. Therefore, over the past decade, scholars examined the extension of the SMC design method to FO nonlinear systems.

Many successful attempts were made by researchers on SMC for the trajectory tracking of FO nonlinear systems. For instance, in [1], a chattering-free SMC method was presented for FO nonlinear systems. The SMC synchronization of FO chaotic systems was studied by [16]. In [3], the consensus tracking of FO multi-agent systems was studied based on SMC. However, these works considered FO nonlinear systems which in their state-space equations the input appears in the same equation as the output. In the cases where the input and output variables are not in the same equation, due to dealing with FO derivatives the control design becomes more challenging.

A huge number of works were published to study the tracking control of FO nonlinear systems with the input and the output state variable appearing in different equations, and addressed a variety of issues by means of various strategies including SMC method. For example, an integral SMC design method was introduced by [9] and a chattering-free one in [10]. An adaptive observer-based control law via a backstepping scheme was suggested in [28] for systems with disturbances and for large-scale systems with unknown parameters and additive disturbances in [5]. In the work of [8], multi-input systems were considered using SMC. The consensus control of multi-agent systems subject to coupling nonlinearities and actuator failures using adaptive control was studied by [7]. In [22], neuro-fuzzy network systems were employed to deal with unknown nonlinear terms, and dynamic surface control (DSC) scheme was constructed to overcome the problem of explosion of complexity caused by the traditional backstepping design. In the work of [23], adaptive neural network tracking control with prescribed performance demands was considered where a FO command filter was adapted to remove the problem of explosion. In [34], adaptive fuzzy decentralized control was utilized to deal with unknown nonlinear functions and unmeasurable states for large-scale systems. Event-triggered adaptive tracking control strategy was applied by [13] to deal with states constraints and dead-zone input. The synchronization of two chaotic systems with disturbance using a fuzzy neural network model and adaptive SMC was considered by [27]. In the article of [36], uncertain systems with multiple mismatched disturbances was investigated using SMC. Systems with input delay were tackled in [30] by using backstepping DSC technology and neural network. Nevertheless, all these works are applicable to a small class of FO nonlinear systems formed as

1 $$\begin{aligned} {\left\{ \begin{array}{ll} D^{{\alpha _i}}{x_i} = {g_i}{x_{i + 1}} ;i = 1,2, \ldots ,n - 1, \\ D^{{\alpha _n}}{x_n} = {f_n} + {g_n}u, \\ y = {x_1}, \\ \end{array}\right. } \end{aligned}$$

where $g_i\in \mathbb {R}$ is a constant for $i=1,2,\ldots ,n-1$ ($D^{{\alpha _i}}$, $x_i$, *u*, *y*, and $f_n$ represent the $\alpha _i$-th FO derivative operator, state variable, input, output, and a function of state variables, respectively, where $\alpha _i\in \mathbb {R}$). In the literature of FO nonlinear systems, this type of systems are referred to as strict-feedback systems [22], while if $g_i$ is a function of the time or state variables, they are referred to as nonstrict-feedback systems [32].

The fundamental challenge in SMC of FO nonstrict-feedback systems is that in the design process, where the sliding surface is a function of the error between the output and the reference input, FO derivative expressions appear as $D^{\alpha _{i+1}}[g_ix_{i+1}]$. In the case of $\alpha _{i+1}=1$, using the Leibniz rule, $D^{\alpha _{i+1}}[g_i x_{i+1}]$ can be easily calculated analytically, comprised of only two terms, and therefore, the classic SMC can be utilized straightforwardly. However, in the case where $0<\alpha _{i+1}<1$ holds, $D^{\alpha _{i+1}}[g_ix_{i+1}]$, according to the Leibniz rule for FO derivative operators, includes an infinite number of terms, which makes the SMC law design challenging [20, 2 of Section 1.1]. Because of this challenge few research works addressed SMC of FO nonstrict-feedback systems. Only in [31], a SMC design was presented for a class of these systems via designing sliding surfaces for each equation of the state-space equations. Besides SMC method, the tracking control of some class of these systems was studied using adaptive control in [20], using adaptive fuzzy control in [29, 32], and using adaptive neural network in [18, 26]. However, all these works did not actually solve the aforementioned challenge, but they used another technique to avoid facing the challenge. In these works, the control law was obtained by designing one virtual input for each single equation, of *n* equations in (1), in a backstepping recursive design algorithm. Nonetheless, this methodology leads to the complexity of the design procedure as well as a large number of design parameters. The number of design parameters dramatically increases with small increase in the number of equations, *n*, which causes the adjustment of the parameters for achieving a desired tracking performance to be very cumbersome. Moreover, the methodologies presented in these works are applicable to the systems with either integer-order or FO dynamics, but not to the systems with a mixture of integer-order and FO dynamics.

Regarding the above discussion, SMC of FO nonstrict-feedback nonlinear systems using the Leibniz rule is an unsolved problem. For a class of these systems a solution is given in this article. For this goal, using the Leibniz rule for the FO derivative, it is proved that only one of the infinite terms resulting from $D^{\alpha _{i+1}}[g_ix_{i+1}]$ is needed to design a SMC law. On the foundation of this point, an algorithm is introduced to design the controller for reference tracking. Afterwards, it is shown that the algorithm has a closed-form solution which presents a simple, straightforward tool to the designer to obtain the controller. The solution has significantly less design parameters than other approaches in the literature do, and also is applicable to the systems with integer-order and/or FO dynamics. Stability and finite-time convergence of the control law are also demonstrated. Finally, the effectiveness of the offered SMC is illustrated via a numerical example coming from a real system.

The rest of the article is organized as follows. Section 2 introduces preliminaries. Section 3 presents the main results. A numerical example and conclusion are given in Sects. 4 and 5, respectively.

## Preliminaries

In this article, $\mathbb {R}$ denotes the set of real numbers, and $\lceil \alpha \rceil$ stands for the smallest integer which is not less than $\alpha$ for any $\alpha \in \mathbb {R}$. For an arbitrary function such as *f*(*t*) its Laplace transform is shown by $\mathcal {L}\{{f(t)}\} = F(s)$. The expresions $\sum \nolimits _{k = {k_1}}^{{k_2}} f_k$ and $\prod \nolimits _{k = {k_1}}^{{k_2}} f_k$ are defined for $k_1\le k_2$. In case $k_1>k_2$ holds, assume $\sum \nolimits _{k = {k_1}}^{{k_2}} f_k =0$ and $\prod \nolimits _{k = {k_1}}^{{k_2}} f_k =1$.

The Caputo definition, the most important in applications, is used for the FO derivatives throughout this article. Suppose $\alpha \in \mathbb {R}$. According to [19, pp. 51,79], the FO integral of an arbitrary function, namely $f:[t_0,\infty )\rightarrow \mathbb {R}$, is defined as

2 $$\begin{aligned} {}_{t_0}D_t^{ - \alpha }f\!\left( t \right) \triangleq \frac{1}{{\varGamma \!\left( \alpha \right) }}\int _{t_0}^t {{{\left( {t - \tau } \right) }^{\alpha - 1}}f\!\left( \tau \right) d\tau } ,\alpha > 0, \end{aligned}$$

where $\varGamma$ stands for the Gamma function. If there exists the $\lceil \alpha \rceil$-th order derivative of *f*(*t*), the Caputo FO derivative of *f*(*t*) is defined as${}_{t_0}D_t^\alpha$ throughout this article represents the Caputo integral and derivative operator of the $\alpha$-th order on $[t_0,t]$ for $\alpha <0$ and $\alpha \ge 0$, respectively.

Some properties of the Caputo FO derivative operator is mentioned in the following lemma, which will be used for calculations in the next section.

### Lemma 1

${}_{t_0}D_t^\alpha$ where $\alpha \in \mathbb {R}$ is a linear operator [4, p. 58]. Moreover, for an arbitrary function such as *f*(*t*), the relation ${}_{t_0}D_t^\alpha {}[{}_{t_0}D_t^{-\alpha }f(t)]=f(t)$ holds for $\alpha \ge 0$ [4, p. 53].

It is notable that for $\alpha _1\ge 0$ and $\alpha _2\ge 0$ the equation ${}_{t_0}D_t^{\alpha _2}[{}_{t_0}D_t^{\alpha _1}f(t)]={}_{t_0}D_t^{\alpha _1+\alpha _2}f(t)$ does not hold generally for the Caputo derivative definition, while some works [12] used this relation (see a counterexample in [11]). The equation is valid in particular cases, namely, when $\alpha _1,\alpha _1+\alpha _2\in [l-1,l]$ holds where *l* is a non-negative integer [4, p. 56]. Therefore, the following notations are introduced in order to be used later.

$$\begin{aligned} {}_{t_0}D_t^{{\alpha _j}|{\alpha _i}}f\!\left( t \right) \triangleq \left\{ {\begin{array}{*{20}{l}} {f\!\left( t \right) ,}&{}{i > j}, \\ {{}_{t_0}D_t^{{\alpha _i}}f\!\left( t \right) ,}&{}{i = j}, \\ {{}_{t_0}D_t^{{\alpha _j}}\left[ {{}_{t_0}D_t^{{\alpha _i}}f\!\left( t \right) } \right] ,}&{}{i < j.} \end{array}} \right. \end{aligned}$$

$$\begin{aligned}&{}{_{t_0}}D_t^{{\alpha _{i + n}}| \cdots |{\alpha _{i + 1}}|{\alpha _i}}f\left( t \right) \\&\quad \triangleq {}{_{t_0}}D_t^{{\alpha _{i + n}}}\left[ { \cdots {}{_{t_0}}D_t^{{\alpha _{i + 1}}}\left[ {{}{_{t_0}}D_t^{{\alpha _i}}f\left( t \right) } \right] } \right] . \end{aligned}$$

Consider the incommensurate FO nonlinear systems described as

3 $$\begin{aligned} \left\{ \begin{aligned}&{}_{t_0}D_t^{{\alpha ' _p}}{x'_p}\!\left( t \right) = {f'_p}\!\left( {t,X'} \right) \\&\qquad + {\varDelta ' _p}\!\left( {t,X'} \right) ,p = 1,2, \ldots ,n' - 1 , \\&{}_{t_0}D_t^{{\alpha ' _{n'}}}{x'_{n'}}\!\left( t \right) = {f'_{n'}}\!\left( {t,X'} \right) \\&\qquad + {g'_{n'}}\!\left( {t,X'} \right) u\!\left( t \right) + {\varDelta ' _{n'}}\!\left( {t,u\!\left( t \right) ,X'} \right) , \\&y\!\left( t \right) = {c_y}{x'_q}\!\left( t \right) ,q \in \left\{ {1,2, \ldots ,n'} \right\} , \\ \end{aligned} \right. \end{aligned}$$

where $0<\alpha '_r\le 1$ holds, ${x'_r}(t)$, *u*(*t*), and *y*(*t*) belong to $\mathbb {R}$, and are the state variable, input, and output, respectively, $X' \triangleq {[{x'_1(t)},{x'_2(t)}, \ldots ,{x'_{n'}(t)}]^T}$, $0\ne c_y\in \mathbb {R}$ is a constant, $\varDelta '_{r}$ represents unknown terms, and $f'_{r}$ and $g'_{n'}$ are known functions where $r=1,2,\ldots ,n'$.

This article deals with those of systems in (3) which, using the appropriate change of the subscripts of $x'_1(t),\alpha '_1,x'_2(t),$ $\alpha '_2,\ldots ,x'_{n'}(t),\alpha '_{n'}$ and denoting them with $x_1(t),\alpha _1,x_2(t),\alpha _2,$ $\ldots ,x_{n'}(t),\alpha _{n'}$, can be reformed as a class of FO nonstrict-feedback nonlinear systems shown as

4a $$\begin{aligned}&{\left\{ \begin{aligned}&{}^{}_{t_0}D_t^{{\alpha _i}}{x_i}\!\left( t \right) \\&= \left\{ {\begin{array}{*{20}{l}} {f_i^{i + 1}\!\left( {t,{X_{i + 1}}} \right) + {\varDelta _i}\!\left( {t,X} \right) ,}&{}{{\alpha _{i + 1}} = 1}, \\ {f_i^i\!\left( {t,{X_i}} \right) + g_i^i\!\left( {t,{X_i}} \right) {x_{i + 1}} \!\left( t\right) }&{}\\ {+ {\varDelta _i}\!\left( {t,X} \right) ,}&{}{0< {\alpha _{i + 1}} < 1} , \end{array}} \right. \\ \qquad \qquad \quad&i = 1,2, \ldots ,n - 1 , \\ {}_{t_0}D_t^{{\alpha _n}}{x_n}\!\left( t \right)&= {f_n}\!\left( {t,X} \right) + {g_n}\!\left( {t,X} \right) u\!\left( t \right) \\&\qquad + {\varDelta _n}\!\left( {t,u\!\left( t\right) ,X} \right) , \\&y\!\left( t \right) = c_y {x_1}\!\left( t \right) , \\ \end{aligned} \right. }\end{aligned}$$

4b $$\begin{aligned}&{}_{t_0}D_t^{{\alpha _j}}{x_j}\!\left( t \right) = {f'_j}\!\left( {t,X} \right) \nonumber \\&\qquad + {\varDelta '_j}\!\left( {t,X} \right) , j = n + 1,n + 2, \ldots ,n', \end{aligned}$$

for which Assumptions 1 and 2 must be met, and if there are more than one choice, the one with the minimum *n* is chosen, where ${X_k} \triangleq [{x_1(t)},{x_2(t)}, \ldots ,{x_k(t)}]^T$ and $X \triangleq [{x_1(t)},{x_2(t)},$ $\ldots , {x_n(t)}]^T$. The known functions ${f_i^{i+1}},{f_i^i},{g_i^{i}}: [{t_0},\infty ) \times \varOmega \rightarrow \mathbb {R}$ are piecewise continuous in $t\in [t_0,\infty )$, and their derivatives exist and are bounded in $X\in \varOmega \subseteq \mathbb {R}^n$ for $i=1,2,\ldots ,n-1$. Moreover, the known functions ${f_n},{g_n},{f'_j}: [{t_0},\infty )\times \varOmega \rightarrow \mathbb {R}$ are piecewise continuous in $t\in [t_0,\infty )$ and locally Lipschitz in $X\in \varOmega \subseteq \mathbb {R}^n$ where $j=n+1,n+2,\ldots ,n'$. In the literature, e.g. [5, 18, 26, 32], similar assumptions such as the smoothness of ${f_i^{i+1}}, {f_i^i},$ and ${g_i^{i}}$ are requirements which imply continuity and local libschitzness. Without loss of generality, assume the system has an equilibrium point at the origin which is included by $\varOmega$, and the subsystem in (4b) is Mittag-Leffler stable in $\varOmega$ (this can be examined with theorems presented by, e.g., [33] and [2]). $\varDelta _k$ represents lumped disturbances for $k=1,2,\ldots ,n$ which will be determined as described in Remark 1 in the following. To avoid clutter, the following definitions are used throughout the article for $i=1,2,\ldots ,n-1$.

$$\begin{aligned} x_i \triangleq x_i\left( t\right) , u \triangleq u\left( t\right) , y \triangleq y\left( t\right) , \end{aligned}$$

$$\begin{aligned} f_i^{i + 1} \triangleq {f_i}(t,{X_{i + 1}}), f_i^i \triangleq {f_i}(t,{X_i}), \end{aligned}$$

$$\begin{aligned} g_i^{i } \triangleq {g_i}(t,{X_{i}}), {\varDelta _i} \triangleq {\varDelta _i}(t,X), \end{aligned}$$

$$\begin{aligned} {f_n} \triangleq {f_n}(t,X), {g_n} \triangleq {g_n}(t,X), {\varDelta _n} \triangleq {\varDelta _n}(t,u,X). \end{aligned}$$

The goal is to present a SMC design method for the output of the system in (4) to track the desired reference input, $y_d(t)$. The three following fundamental assumptions are considered concerning with the system in (4).

### Assumption 1

$g_n\ne 0$ and $\rho _i\ne 0$ hold in $X\in \varOmega$ for $i=1,2,\ldots ,n-1$ where

5 $$\begin{aligned} {\rho _i} \triangleq {\rho _i}\!\left( {t,{X_{i + 1}}} \right) \triangleq \left\{ {\begin{array}{*{20}{l}} {\frac{{\partial f_i^{i + 1}}}{{\partial {x_{i + 1}}}},}&{}{{\alpha _{i + 1}} = 1}, \\ {g_i^{i},}&{}{0< {\alpha _{i + 1}} < 1}. \end{array}} \right. \end{aligned}$$

### Assumption 2

Define

6 $$\begin{aligned} {h_\varDelta } \triangleq \sum \limits _{k = 1}^n {{}_{t_0}D_t^{{\alpha _n}| \cdots |{\alpha _{k + 1}}}\left[ {{\varDelta _k}\prod \limits _{r = 1}^{k - 1} {{\rho _r}} } \right] }. \end{aligned}$$

Given $\gamma \in (0,1]$ and $\beta \in (0,\infty )$, it is assumed that $\varDelta _k$ is sufficiently smooth and bounded for $k=1,2,\ldots ,n$ such that there is a known, finite $c_\varDelta$ which meets

7 $$\begin{aligned} | {c_y{}_{t_0}D_t^{ \gamma | \beta }{h_\varDelta }}| \le {c_\varDelta }. \end{aligned}$$

### Assumption 3

It is assumed that $y_d(t)$ is sufficiently smooth such that ${}_{t_0}D_t^{\gamma |\beta |{\alpha _n}| \cdots |{\alpha _{2}}|{\alpha _1}}{y_d(t)}$ exists and is bounded.

It is notable that the above assumptions are equivalent to similar, conventional assumptions in the literature related to FO nonlinear systems; see, e.g., [31, Assumption 2] and [8, Assumptions 1 and 3]. Assumptions 1-3 together avoid the singularity of the control signal, as will be shown in the next section.

### Remark 1

$\varDelta _k$ in (4) represents lumped disturbances including known internal disturbances, unknown external disturbances, and unknown unmodeled dynamics for $k=1,2,\ldots ,n$. To transform the state-space equations of a system from (3) to (4), those terms which can not be considered as part of $f_k^{k+1}$, $f_k^k$, or $g_k^{k}$ can be added to $\varDelta '_k$; these terms are called here known internal disturbances. Therefore, $\varDelta _k$ is the summation of $\varDelta '_k$ and the terms added as internal disturbances. In this case, Assumption 2 must be met by the new obtained $\varDelta _k$.

The stability definition used throughout this article is the Mittag-Leffler stability [33]. The Lyapunov-based conditions for the stability of the system in (4) is presented in the following lemma.

### Lemma 2

[33] Let $X_{n0}\triangleq [x_{10},x_{20},$ $\ldots , x_{n0}]^T=0$ be an equilibrium point for the system in (4) and $\varOmega \subseteq \mathbb {R}^n$ be a domain containing the origin. Let $V: [{0},\infty )\times \varOmega \rightarrow \mathbb {R}$ be a continuously differentiable function in $t\in [0,\infty )$ and locally Lipschitz in $X\in \varOmega$ such that

$$\begin{aligned} {b_1}{\left\| X \right\| ^{{b_4}}} \le V\!\left( {t,X} \right) \le {b_2}{\left\| X \right\| ^{{b_4}{b_5}}}, \end{aligned}$$

$$\begin{aligned} \begin{aligned} {}_{0}D_t^\gamma V\!\left( {t,X} \right) \le - {b_3}{\left\| X \right\| ^{{b_4}{b_5}}}, \end{aligned} \end{aligned}$$

where $\gamma \in (0,1]$, $b_1$, $b_2$, $b_3$, $b_4$, and $b_5$ are arbitrary positive constant and ||.|| denotes an arbitrary norm. Then, $X_{n0}$ is asymptotically Mittag-Leffler stable.

The following lemma is helpful to check the conditions in the previous lemma.

### Lemma 3

[2] Let $\gamma \in (0,1]$ and $x(t)\in \mathbb {R}$ be a continuous and differentiable function. It follows that

$$\begin{aligned} \frac{1}{2}{}_{t_0}D_t^\gamma {x^2}\!\left( t \right) \le x\!\left( t \right) {}_{t_0}D_t^\gamma x\!\left( t \right) . \end{aligned}$$

The Leibniz rule for FO derivatives of the product of two functions is stated as follows.

### Lemma 4

[p. 59 of [4]] Let $0<\alpha <1$ hold, and assume that *f* and *g* are analytic on $(t_0-h,t_0+h)$. Then,

8 $$\begin{aligned} {}_{t_0}D_t^\alpha \left[ {f\!\left( t \right) g\!\left( t \right) } \right] =&\frac{{{{\left( {t - t_0} \right) }^{ - \alpha }}}}{{\varGamma \!\left( {1 - \alpha } \right) }}f\!\left( {{t_0}} \right) \left( {g\!\left( t \right) - g\!\left( {{t_0}} \right) } \right) \nonumber \\&+ \sum \limits _{k = 0}^\infty {\left( {\begin{array}{*{20}{c}} \alpha \\ k \end{array}} \right) {}_{t_0}D_t^{\alpha - k}f\!\left( t \right) {}_{t_0}D_t^k g\!\left( t \right) }. \end{aligned}$$

The following lemma will be used for the Laplace transform.

### Lemma 5

[p. 134 of [4]] Assume $\mathcal {L}\{{f(t)}\} = F(s)$ and that $f:[0,\infty )\rightarrow \mathbb {R}$ is such that its Laplace transform exists on $[\hat{t},\infty )$ with some $\hat{t}\in \mathbb {R}$. Let $\alpha >0$. Then, for $t>\max \{0,\hat{t}\}$ we have

9 $$\begin{aligned} \mathcal {L}\left\{ {{}{_0}D_t^{ - \alpha }f\!\left( t \right) } \right\}= & {} \frac{1}{{{s^\alpha }}}F\!\left( s \right) , \end{aligned}$$

10

## Main results

In this section, first, an algorithm is given to design a SMC law for the output of the system in (4) to track a desired reference input. Then, a closed-form solution for the algorithm outcome is presented which gives the designer a user-friendly tool to obtain the controller.

The following two lemmas are needed to introduce the design algorithm of SMC.

### Lemma 6

Consider the parameters defined for the system in (4). Assume $h_1^{i}\triangleq h_1(t,X_{i})$ and $h_2^{i}\triangleq h_2(t,\varDelta _i,X_{i})$ are arbitrary differentiable functions where $i=1,2,\ldots ,{n-1}$. Then, it follows that

11 $$\begin{aligned} {\left\{ {\begin{array}{*{20}{l}} {{}_{t_0}D_t^{{\alpha _{i + 1}}}\left[ {f_i^{i + 1}h_1^i + h_2^i} \right] }&{}\\ {\quad = {}_{t_0}D_t^{{\alpha _{i + 1}}}{x_{i + 1}}\frac{{\partial f_i^{i + 1}}}{{\partial {x_{i + 1}}}}h_1^i}&{}\qquad {{\alpha _{i + 1}} = 1}, \\ \qquad \qquad + \Bigg \{ {{}_{t_0}D_t^{{\alpha _{i + 1}}}\left[ {f_i^{i + 1}h_1^i} \right] } &{} \\ \qquad \qquad {- {}_{t_0}D_t^{{\alpha _{i + 1}}}{x_{i + 1}}\frac{{\partial f_i^{i + 1}}}{{\partial {x_{i + 1}}}}h_1^i} \Bigg \}&{}{} \\ { + \left\{ {{}_{t_0}D_t^{{\alpha _{i + 1}}}h_2^i} \right\} ,}&{}{} \\ {{}_{t_0}D_t^{{\alpha _{i + 1}}}\left[ {{x_{i + 1}}g_i^{i}h_1^i + h_2^i} \right] } &{} \\ {= {}_{t_0}D_t^{{\alpha _{i + 1}}}{x_{i + 1}}g_i^{i}h_1^i}&{}\qquad {0< {\alpha _{i + 1}} < 1}, \\ + \Bigg \{ {{}_{t_0}D_t^{{\alpha _{i + 1}}}\left[ {{x_{i + 1}}g_i^{i}h_1^i} \right] }&{} \\ {\quad - {}_{t_0}D_t^{{\alpha _{i + 1}}}{x_{i + 1}}g_i^{i}h_1^i} \Bigg \}&{}{} \\ { + \left\{ {{}_{t_0}D_t^{{\alpha _{i + 1}}}h_2^i} \right\} ,}&{}{} \end{array}} \right. } \end{aligned}$$

where the terms in the curly brackets, $\{.\}$, do not include ${}_{t_0}D_t^{{\alpha _{i + 1}}}{x_{i + 1}}$ and any derivative of $x_{i+1}$.

### Proof

We notice that since $h_1^i$ and $h_2^i$ do not have $x_{i+1}$, ${}_{t_0}D_t^{{\alpha _{i + 1}}}h_1^i$ and ${}_{t_0}D_t^{{\alpha _{i + 1}}}h_2^i$ do not include ${}_{t_0}D_t^{{\alpha _{i + 1}}}x_{i+1}$. For the case of $\alpha _{i+1}=1$, we have

12 $$\begin{aligned} {}_{t_0}D_t^{{\alpha _{i + 1}}}&\left[ {f_i^{i + 1}h_1^i + h_2^i} \right] \nonumber \\ =&\sum \limits _{k = 1}^{i + 1} {{}_{t_0}D_t^{{\alpha _{i + 1}}}{x_k}\frac{{\partial \left[ {f_i^{i + 1}h_1^i} \right] }}{{\partial {x_k}}}} + \left\{ {{}_{t_0}D_t^{{\alpha _{i + 1}}}h_2^i} \right\} \nonumber \\ =&\left\{ {\sum \limits _{k = 1}^i {{}_{{t_0}}D_t^{{\alpha _{i + 1}}}{x_k}\frac{{\partial \left[ {f_i^{i + 1}h_1^i} \right] }}{{\partial {x_k}}}} } \right\} \nonumber \\&+ {}_{{t_0}}D_t^{{\alpha _{i + 1}}}{x_{i + 1}}\frac{{\partial \left[ {f_i^{i + 1}h_1^i} \right] }}{{\partial {x_{i + 1}}}} + \left\{ {{}_{{t_0}}D_t^{{\alpha _{i + 1}}}h_2^i} \right\} \nonumber \\&= \left\{ \underbrace{\sum \limits _{k = 1}^i {{}_{t_0}D_t^{{\alpha _{i + 1}}}{x_k}\frac{{\partial \left[ {f_i^{i + 1}h_1^i} \right] }}{{\partial {x_k}}}} + {{{}_{t_0}D_t^{{\alpha _{i + 1}}}{x_{i + 1}}\frac{{\partial f_i^{i + 1}}}{{\partial {x_{i + 1}}}}h_1^i}}}_{{}_{t_0}D_t^{{\alpha _{i + 1}}}\left[ {f_i^{i + 1}h_1^i} \right] } \right. \nonumber \\&\left. - {{{}_{t_0}D_t^{{\alpha _{i + 1}}}{x_{i + 1}}\frac{{\partial f_i^{i + 1}}}{{\partial {x_{i + 1}}}}h_1^i}} \right\} \nonumber \\&\qquad + {}_{t_0}D_t^{{\alpha _{i + 1}}}{x_{i + 1}}\frac{{\partial f_i^{i + 1}}}{{\partial {x_{i + 1}}}}h_1^i \nonumber \\&+ \left\{ {{}_{t_0}D_t^{{\alpha _{i + 1}}}h_2^i} \right\} . \end{aligned}$$

In (12), obviously since the last two terms in the curly brackets cancel each other, the curly brackets do not include ${}_{t_0}D_t^{{\alpha _{i + 1}}}x_{i+1}$ and any derivative of $x_{i+1}$. For the case of $0<\alpha _{i+1}<1$, using Lemma 1, we have

13 $$\begin{aligned} {}_{t_0}D_t^{{\alpha _{i + 1}}}\left[ {{x_{i + 1}}g_i^{i}h_1^i + h_2^i} \right]= & {} {}_{t_0}D_t^{{\alpha _{i + 1}}}\left[ {{x_{i + 1}}g_i^{i}h_1^i} \right] \nonumber \\{} & {} + \left\{ {{}_{t_0}D_t^{{\alpha _{i + 1}}}h_2^i} \right\} . \end{aligned}$$

Defining $f(t)\triangleq x_{i+1}$, $g(t)\triangleq g_i^{i}h_1^i$, and $\alpha \triangleq \alpha _{i+1}$, one can check that among the infinite terms on the right side of (8) only the term ${}_{t_0}D_t^{\alpha _{i+1}}x_{i+1}g_i^{i}h_1^i$ includes ${}_{t_0}D_t^{\alpha _{i+1}}x_{i+1}$. Therefore, the first term on the right side of (13) can be rewritten as

$$\begin{aligned}{} & {} \left\{ {{}_{t_0}D_t^{{\alpha _{i + 1}}}\left[ {{x_{i + 1}}g_i^{i}h_1^i} \right] - {}_{t_0}D_t^{{\alpha _{i + 1}}}{x_{i + 1}}g_i^{i }h_1^i} \right\} \nonumber \\{} & {} + {}_{t_0}D_t^{{\alpha _{i + 1}}}{x_{i + 1}}g_i^{i }h_1^i, \end{aligned}$$

where the terms in the curly brackets do not include ${}_{t_0}D_t^{\alpha _{i+1}}x_{i+1}$ and any derivative of $x_{i+1}$ because the term ${}_{t_0}D_t^{\alpha _{i+1}}x_{i+1}g_i^{i }h_1^i$ incorporated in ${}_{t_0}D_t^{{\alpha _{i + 1}}}[ {{x_{i + 1}}g_i^{i}h_1^i} ]$ cancels the second term in the curly brackets. $\square$

In designing a SMC law in Theorem 1, one will see that we need to calculate ${}_{t_0}D_t^{{\alpha _{i + 1}}}[ f_i^{i + 1}h_1^i + h_2^i ]$ or ${}_{t_0}D_t^{{\alpha _{i + 1}}}[ {x_{i + 1}}g_i^{i}h_1^i + h_2^i ]$, and then replace ${}_{t_0}D_t^{\alpha _{i+1}}x_{i+1}$ with the system dynamics in (4a). However, according to (8), ${}_{t_0}D_t^{{\alpha _{i + 1}}}[ f_i^{i + 1}h_1^i + h_2^i ]$ and ${}_{t_0}D_t^{{\alpha _{i + 1}}}[ {x_{i + 1}}g_i^{i}h_1^i + h_2^i ]$ produce an infinite number of terms, among which we do not know which terms include ${}_{t_0}D_t^{\alpha _{i+1}}x_{i+1}$. Lemma 6 reveals that only one term out of those infinite terms depends on ${}_{t_0}D_t^{\alpha _{i+1}}x_{i+1}$, and extracts that single term outside of the curly brackets, as in (11).

### Lemma 7

Assume $\mathcal {L}\{{f(t)}\} = F(s)$ and that $f:[0,\infty )\rightarrow \mathbb {R}$ is such that its Laplace transform exists on $[\hat{t},\infty )$ with some $\hat{t}\in \mathbb {R}$. Let $0<\alpha _k\le 1$ hold for $k=1,2,\ldots ,n$. Then, for $t>\max \{0,\hat{t}\}$ we have

14 $$\begin{aligned}&\mathcal {L}\left[ {{}{_0}D_t^{{\alpha _n}| \cdots |{\alpha _1}}f\!\left( t \right) } \right] = {s^{\sum \nolimits _{r = 1}^n {{\alpha _r}} }}F\!\left( s \right) \nonumber \\&\quad - \sum \limits _{k = 1}^n {s^{\sum \nolimits _{r = 1}^k {{\alpha _r}} - 1}}{{\left[ {{}{_0}D_t^{{\alpha _n}| \cdots |{\alpha _{k + 1}}}f\!\left( t \right) } \right] }_{t = 0}} . \end{aligned}$$

### Proof

Using (10), one can write

$$\begin{aligned} \mathcal {L}&\left[ {{}{_0}D_t^{{\alpha _n}| \cdots |{\alpha _2}|{\alpha _1}}f\!\left( t \right) } \right] ={s^{{\alpha _1}}}\mathcal {L}\left[ {{}{_0}D_t^{{\alpha _n}| \cdots |{\alpha _2}}f\!\left( t \right) } \right] \\&\qquad - {s^{{\alpha _1} - 1}}{\left[ {{}{_0}D_t^{{\alpha _n}| \cdots |{\alpha _2}}f\!\left( t \right) } \right] _{t = 0}}\\&\quad ={s^{{\alpha _1}}}\Bigg [ {s^{{\alpha _2}}}\mathcal {L}\left[ {{}{_0}D_t^{{\alpha _n}| \cdots |{\alpha _3}}f\!\left( t \right) } \right] \\&\qquad - {s^{{\alpha _2} - 1}}{{\left[ {}{_0}D_t^{{\alpha _n}| \cdots |{\alpha _3}}f\!\left( 0 \right) \right] }_{t = 0}} \Bigg ]\\&\qquad - {s^{{\alpha _1} - 1}}{\left[ {{}{_0}D_t^{{\alpha _n}| \cdots |{\alpha _2}}f\!\left( t \right) } \right] _{t = 0}}\\&\qquad ={s^{{\alpha _1} + {\alpha _2}}}\mathcal {L}\left[ {{}{_0}D_t^{{\alpha _n}| \cdots |{\alpha _3}}f\!\left( t \right) } \right] \\&\qquad - {s^{{\alpha _1} + {\alpha _2} - 1}}{\left[ {{}{_0}D_t^{{\alpha _n}| \cdots |{\alpha _3}}f\!\left( 0 \right) } \right] }_{t = 0} \\ {}&\qquad - {s^{{\alpha _1} - 1}}{\left[ {{}{_0}D_t^{{\alpha _n}| \cdots |{\alpha _2}}f\!\left( t \right) } \right] _{t = 0}}. \end{aligned}$$

From the above equations, it is easy to derive (14). $\square$

The following theorem offers an algorithm to design a SMC law for the system in (4).

### Theorem 1

Assume $y_d\triangleq y_d(t)$ and $e\triangleq e(t)=y-y_d$ are, respectively, the desired output (reference input) and the tracking error for the system in (4) with Assumptions 1, 2, and 3. Suppose $\gamma \in (0,1]$ and $k_s\in (0,\infty )$ are arbitrary values. Define the sliding surface

15 $$\begin{aligned} S\!\left( t\right) \triangleq {}_{t_0}D_t^{\beta |{\alpha _n}| \cdots |{\alpha _{2}}|{\alpha _1}}e + \sum \limits _{l = 0}^{m - 1} {{c_l}{}_{t_0}D_t^{\frac{{l\beta }}{m}|\frac{{l{\alpha _n}}}{m}| \cdots |\frac{{l{\alpha _{2}}}}{m}|\frac{{l{\alpha _1}}}{m}}e}, \end{aligned}$$

where $m\in \{1,2,\ldots \}$ and $\beta \in \mathbb {R}$ are chosen in such a way that

16 $$\begin{aligned}{} & {} \beta \ge 0, \end{aligned}$$

17 $$\begin{aligned}{} & {} \beta + \sum \limits _{k = 1}^n {{\alpha _k}} < 2m \end{aligned}$$

hold, and $c_0,c_1,\ldots ,c_{m-1}\in \mathbb {R}$ are also chosen in such a way that all the roots of

18 $$\begin{aligned} r^m + \sum \limits _{l = 0}^{m-1} {{c_l}{r^l}} = 0, \end{aligned}$$

denoted by $r_l$ for $l=1,2,\ldots ,m$, satisfy the relation

19 $$\begin{aligned} \left| \arg \left( {{r_l}} \right) \right| > \frac{\pi }{{2m}}\left( \beta + {\sum \limits _{k = 1}^n {{\alpha _k}} } \right) . \end{aligned}$$

Then, defining $u_{eq}\triangleq u_{eq}(t)$ and $u_r\triangleq u_r(t)$ as equivalent and reaching inputs, respectively, the closed-loop system is asymptotically stable with the control law

20 $$\begin{aligned} u=u_{eq}+u_r, \end{aligned}$$

where

21 $$\begin{aligned} {u_{eq}}&= - \frac{{{f_n}}}{{{g_n}}} - \frac{\mu }{{{\rho }{g_n}}}{ - \frac{{{}_{t_0}D_t^{ - \beta | - \gamma }\left[ {\sum \limits _{l = 0}^{m - 1} {{c_l}{}_{t_0}D_t^{\gamma |\frac{{l\beta }}{m}|\frac{{l{\alpha _n}}}{m}| \cdots |\frac{{l{\alpha _{2}}}}{m}|\frac{{l{\alpha _1}}}{m}}e} - {}_{t_0}D_t^{\gamma |\beta |{\alpha _n}| \cdots |{\alpha _{2}}|{\alpha _1}}{y_d}} \right] }}{{c_y{\rho }{g_n}}}}, \end{aligned}$$

22 $$\begin{aligned} {u_r}&= \frac{{{}_{t_0}D_t^{ - \beta | - \gamma }\left[ { - {k_s}S\!\left( t\right) - {c_\varDelta }{\text {sign}}\!\left( S\!\left( t\right) \right) } \right] }}{{c_y{\rho }{g_n}}}, \end{aligned}$$

and the functions $\rho \triangleq \rho (t,X)$, $\mu \triangleq \mu (t,X)$, and $h_\varDelta \triangleq h_\varDelta (t,X)$ are obtained as follows. Given $i=1$, replace ${}_{t_0}D_t^{\alpha _i}x_i$ from (4) and then apply ${}_{t_0}D_t^{\alpha _{i+1}}$ using (11). Therefore, one has

$$\begin{aligned} {}_{t_0}D_t^{{\alpha _1}}{x_1} = \left\{ {\begin{array}{*{20}{l}} {f_1^2 + {\varDelta _1},}&{}{{\alpha _2} = 1,} \\ {f_1^1 + g_1^1{x_2} + {\varDelta _1},}&{}{0< {\alpha _2} < 1,} \end{array}} \right. \Rightarrow \end{aligned}$$

23 $$\begin{aligned} {}_{t_0}D_t^{{\alpha _2}|{\alpha _1}}{x_1} = \left\{ {\begin{array}{*{20}{l}} {{}_{t_0}D_t^{{\alpha _2}}\left[ {f_1^2 \!+\! {\varDelta _1}} \right] ,}&{}\!\!{{\alpha _2} \!=\! 1,} \\ {{}_{t_0}D_t^{{\alpha _2}}\left[ {f_1^1 \!+\! g_1^1{x_2} \!+\! {\varDelta _1}} \right] ,}&{}\!\!{0 \!<\! {\alpha _2} \!<\! 1.} \end{array}} \right. \end{aligned}$$

Employing (11), from (23) we get

24 $$\begin{aligned} {}_{t_0}D_t^{{\alpha _2}|{\alpha _1}}{x_1} = {}_{t_0}D_t^{{\alpha _2}}{x_2}w_1^1 + \left\{ {w_2^1} \right\} \end{aligned}$$

where, according to Lemma 6, $w_1^1$ and $w_2^1$ are functions that do not include ${}_{t_0}D_t^{\alpha _{2}}x_{2}$. Similarly, given $i=2$, replace ${}_{t_0}D_t^{\alpha _i}x_i$ in (24) from (4) and then apply ${}_{t_0}D_t^{\alpha _{i+1}}$ using (11). Therefore, one has

$$\begin{aligned}&{}_{t_0}D_t^{{\alpha _3}|{\alpha _2}|{\alpha _1}}{x_1} \\&\quad = \left\{ \!\! {\begin{array}{*{20}{l}} {{}_{t_0}D_t^{{\alpha _3}}\!\left[ {f_2^3 + {\varDelta _2}} \right] w_1^1 + \left\{ {w_2^1} \right\} ,}&{}{{\alpha _3} \!=\! 1,} \\ {{}_{t_0}D_t^{{\alpha _3}}\!\left[ {f_2^2 + g_2^2{x_3} + {\varDelta _2}} \right] w_1^1 + \left\{ {w_2^1} \right\} ,}&{}{0 \!<\! {\alpha _3} \!<\! 1,} \end{array}} \right. \end{aligned}$$

$$\begin{aligned} \Rightarrow {}_{t_0}D_t^{{\alpha _3}|{\alpha _2}|{\alpha _1}}{x_1} = {}_{t_0}D_t^{{\alpha _3}}{x_3}w_1^2 + \left\{ {w_2^2} \right\} \end{aligned}$$

where $w_1^2$ and $w_2^2$ are functions that do not include ${}_{t_0}D_t^{\alpha _{3}}x_{3}$. Keeping performing these steps, the final result for $i=n-1$ will be formed as

25 $$\begin{aligned} {}_{t_0}D_t^{{\alpha _n}| \cdots |{\alpha _{2}}|{\alpha _1}}{x_1} = {\rho }\left( {{f_n} + {g_n}u} \right) + {\mu } + {h_\varDelta }, \end{aligned}$$

from which $\rho$, $\mu$, and $h_\varDelta$ can be extracted, considering that the terms including $\varDelta _1,\varDelta _2,\ldots ,\varDelta _n$ belong to $h_\varDelta$.

### Proof

In the following, first, it is proved that the sliding surface in (15) is stable. Then, it is shown that the trajectory of *e* on the surface converges to the origin asymptotically. For checking the stability of *S*(*t*), the Lyapunov function

26 $$\begin{aligned} V\!\left( S\right) \triangleq \frac{1}{2} S^2\!\left( t\right) \end{aligned}$$

is chosen. Using Lemma 3, one can write

27 $$\begin{aligned} {}_{t_0}D_t^{\gamma }V \!\left( S\right) \le S\!\left( t\right) {}_{t_0}D_t^{\gamma }S\!\left( t\right) . \end{aligned}$$

Using $e=y-y_d$ and $y=c_y x_1$, from (15), it is concluded that

28 $$\begin{aligned} {}_{t_0}D_t^\gamma S\!\left( t\right) =&c_y{}_{t_0}D_t^{\gamma |\beta |{\alpha _n}| \cdots |{\alpha _{2}}|{\alpha _{1}}}{x_1} - {}_{t_0}D_t^{\gamma |\beta |{\alpha _n}| \cdots |{\alpha _{2}}|{\alpha _1}}{y_d} \nonumber \\&+ \sum \limits _{l = 0}^{m - 1} {{c_l}{}_{t_0}D_t^{\gamma |\frac{{l\beta }}{m}|\frac{{l{\alpha _n}}}{m}| \cdots |\frac{{l{\alpha _{2}}}}{m}|\frac{{l{\alpha _1}}}{m}}e}. \end{aligned}$$

The goal of the $n-1$ steps mentioned in the theorem, using (11), is actually to develop the first term on the right side of (28) and to replace ${}_{t_0}D_t^{\alpha _{i+1}}x_{i+1}$, for $i=1,2,\ldots ,n-1$, until a point where ${}_{t_0}D_t^{\alpha _n}x_n$ appears. Therefore, it can be replaced using the dynamics of the system in (4), and consequently *u* appears and can be obtained such that $S(t){}_{t_0}D_t^{\gamma }S(t)<0$ holds. Regarding (11), the terms resulting from applying ${}_{t_0}D_t^{\alpha _{i+1}}$ to its argument include a term which is the product of ${}_{t_0}D_t^{\alpha _{i+1}}x_{i+1}$ and another function that does not include ${}_{t_0}D_t^{\alpha _{i+1}}x_{i+1}$ plus an infinite number of other terms in curly brackets that do not include ${}_{t_0}D_t^{\alpha _{i+1}}x_{i+1}$, either. That is, among these infinite terms resulting from a FO derivative operator only one term is needed here. considering this point and performing the steps mentioned in the theorem it is easy to infer that we expect to get

29 $$\begin{aligned} {}_{t_0}D_t^{{\alpha _n}| \cdots |{\alpha _{2}}|{\alpha _1}}{x_1} =\rho {}_{t_0}D_t^{\alpha _{n}}x_{n}+\mu +h_\varDelta ^{n-1} \end{aligned}$$

in step $n-1$ where $\rho$ and $\mu +h_\varDelta ^{n-1}$ do not include ${}_{t_0}D_t^{\alpha _{n}}x_{n}$ and

30 $$\begin{aligned} h_\varDelta ^{n-1}\triangleq h_\varDelta -\rho \varDelta _n. \end{aligned}$$

Using the dynamics of the system in (4) we have ${}_{t_0}D_t^{\alpha _{n}}x_{n}={f_n} + {g_n}u+\varDelta _n$. By replacing this in (29) and using (30), one gets (25). Substituting (25) into (28) yields

31 $$\begin{aligned} {}_{t_0}D_t^\gamma S\!\left( t\right) =&c_y{}_{t_0}D_t^{\gamma |\beta }\left[ {\rho \left( {{f_n} + {g_n}u} \right) + \mu + {h_\varDelta }} \right] \nonumber \\&\quad - {}_{t_0}D_t^{\gamma |\beta |{\alpha _n}| \cdots |{\alpha _{2}}|{\alpha _1}}{y_d} \nonumber \\&\quad + \sum \limits _{l = 0}^{m - 1} {{c_l}{}_{t_0}D_t^{\gamma |\frac{{l\beta }}{m}|\frac{{l{\alpha _n}}}{m}| \cdots |\frac{{l{\alpha _{2}}}}{m}|\frac{{l{\alpha _1}}}{m}}e}. \end{aligned}$$

In view of Assumptions 1, 2 and 3, (20)–(22) are nonsingular. Substituting (20)–(22) into (31), applying some simplification using Lemma 1 and considering (16) and $\gamma \in (0,1]$, gives

32 $$\begin{aligned} {}_{t_0}D_t^\gamma S\!\left( t\right) = - {k_s}S\!\left( t\right) - {c_\varDelta }{\text {sign}}\!\left( S\!\left( t\right) \right) + c_y{}_{t_0}D_t^{\gamma |\beta }{h_\varDelta } . \end{aligned}$$

Substituting (32) into (27) and utilizing (7) gives

33 $$\begin{aligned} {}_{t_0}D_t^\gamma V\!\left( S\right) \le&- {k_s}{S^2\!\left( t\right) } \underbrace{ - {c_\varDelta }\left| S\!\left( t\right) \right| +c_y{}_{t_0}D_t^{\gamma |\beta }{h_\varDelta } S\!\left( t\right) }_{ \text {non-positive}} \nonumber \\ \le&- {k_s}{S^2\!\left( t\right) } < 0 . \end{aligned}$$

Therefore, based on Lemma 2, the sliding surface *S*(*t*) is stable. In the following, it is shown that after the trajectory of *e* reaches the surface at the reach time, $t=t_r$, it converges to the origin asymptotically. Considering $S(t_r)=0$ and tacking the Laplace transform of (15) using Lemma 7 gives

$$\begin{aligned}&{s^{\beta + \sum \nolimits _{k = 1}^n {{\alpha _k}} }}E\!\left( s \right) \\&\qquad + \sum \limits _{l = 0}^{m - 1} {{c_l}{s^{\frac{l}{m}\left( {\beta + \sum \nolimits _{k = 1}^n {{\alpha _k}} } \right) }}E\!\left( s \right) } - N\!\left( {s,e\!\left( {{t_r}} \right) } \right) = 0 \Rightarrow \end{aligned}$$

34 $$\begin{aligned} E\!\left( s \right) = \frac{{N\!\left( {s,e\!\left( {{t_r}} \right) } \right) }}{{{s^{\beta + \sum \nolimits _{k = 1}^n {{\alpha _k}} }} + \sum \limits _{l = 0}^{m - 1} {{c_l}{s^{\frac{l}{m}\left( {\beta + \sum \nolimits _{k = 1}^n {{\alpha _k}} } \right) }}} }}. \end{aligned}$$

where $N(s,e(t_r)$ is a FO polynomial. According to [15, pp. 19-22], *E*(*s*) in (34) is asymptotically stable if and only if the roots of

35 $$\begin{aligned} {\lambda ^{ {\beta + \sum \nolimits _{k = 1}^n {{\alpha _k}} } }} + \sum \limits _{l = 0}^{m - 1} {{c_l}{\lambda ^{\frac{l}{m}\left( {\beta + \sum \nolimits _{k = 1}^n {{\alpha _k}} } \right) }}} = 0 \end{aligned}$$

on the principal Riemann sheet, denoted by $\lambda _l$ for $l=1,2,\ldots ,m$, satisfy the relation

$$\begin{aligned} \left| \arg \left( {{\lambda _l}} \right) \right| > \frac{\pi }{{2}}, \end{aligned}$$

which is equivalent to that the roots of (18) satisfy the relation in (19) where $r \triangleq {\lambda ^{\frac{1}{m}(\beta + \sum \nolimits _{k = 1}^n {{\alpha _k}} )}}$. Moreover, supposing that $\arg (\theta )\in (-\pi ,\pi ]$ holds for a $\theta$ on the complex plane, (17) guarantees that the area characterized in (19) is not null. Therefore, *E*(*s*) is stable and the trajectory of *e* on it converges to the origin asymptotically. $\square$

In the following corollary, it is shown that the trajectory of *e* reaches the sliding surface in finite time.

### Corollary 1

Under the SMC designed in Theorem 1, the trajectory of the error reaches the sliding surface in finite time.

### Proof

From (33), it is deduced that there exists a finite, positive constant such as $c_v>0$ such that

36 $$\begin{aligned} {}_{t_0}D_t^\gamma V\!\left( S\right) \le -c_v. \end{aligned}$$

Considering $t_0=0$, from (36) and (26), it is inferred that there is a function such as $h_v(t)\ge 0$ such that

37 $$\begin{aligned} 0.5 {}{_0}D_t^\gamma S^2\!\left( t\right) = -c_v-h_v\!\left( t\right) . \end{aligned}$$

Taking the Laplace transform of (37) using (10), one has

$$\begin{aligned} {s^\gamma }\mathcal {L}\left\{ {S^2\!\left( t\right) } \right\} - {s^{\gamma - 1}}S^2\!\left( 0\right) = - \frac{{{c_v}}}{s} - {H_v}\!\left( s \right) \Rightarrow \end{aligned}$$

38 $$\begin{aligned} \mathcal {L}\left\{ {S^2\!\left( t\right) } \right\} = \frac{{S^2\!\left( 0\right) }}{s} - \frac{{{c_v}}}{{{s^{\gamma + 1}}}} - \frac{{{H_v}\!\left( s \right) }}{{{s^\gamma }}}. \end{aligned}$$

Regarding that ${\mathcal {L}^{ - 1}}\{ 1/{s^{\gamma + 1}}\} = {t^\gamma }/\varGamma (\gamma + 1)$ [15, p. 27] and also using (9), from (38) one gets

39 $$\begin{aligned} S^2\!\left( t\right) = S^2\!\left( 0\right) - \frac{{{c_v}{t^\gamma }}}{{\varGamma \!\left( {\gamma + 1} \right) }} - {}{_0}D_t^{ - \gamma }{h_v}\!\left( t \right) . \end{aligned}$$

Since $h_v(t)\ge 0$ holds, considering (2), we conclude that ${}{_0}D_t^{ - \gamma }{h_v}\left( t \right) \ge 0$ holds. Moreover, denoting the reaching time with $t_r$, $S(t_r)=0$ holds. Hence, (39) can result in

$$\begin{aligned} {S^2\!\left( 0\right) } - \frac{{{c_v}t_r^\gamma }}{{\varGamma \!\left( {\gamma + 1} \right) }} \ge 0 \Rightarrow {t_r} \le {\left( {\frac{{S^2\!\left( 0\right) \varGamma \!\left( {\gamma + 1} \right) }}{{{c_v}}}} \right) ^{\frac{1}{\gamma }}}. \end{aligned}$$

Therefore, the reaching time, $t_r$, is finite. $\square$

Closed-form solutions for the functions $\rho$, $\mu$, and $h_\varDelta$, used in the SMC law in (20)–(22), are obtained in the following theorem.

### Theorem 2

The functions $\rho$, $\mu$, and $h_\varDelta$, used in the SMC law in (20)–(22) can be obtained with the relations

40 $$\begin{aligned} \rho = \prod \limits _{i = 1}^{n - 1} {{\rho _i}}, \end{aligned}$$

41 $$\begin{aligned} \mu = \sum \limits _{i = 1}^{n - 1} {{}_{t_0}D_t^{{\alpha _n}| \cdots |{\alpha _{i + 2}}}{\mu _i}}, \end{aligned}$$

and (6), where $\rho _i$ was defined in (5), and

$$\begin{aligned}&\mu _i \triangleq {\mu _i}\!\left( {t,{X_{i + 1}}} \right) \triangleq \\&\left\{ {\begin{array}{*{20}{l}} {{}_{t_0}D_t^{{\alpha _{i + 1}}}\left[ {f_i^{i + 1}\prod \limits _{r = 1}^{i - 1} {{\rho _r}} } \right] }&{}{{\alpha _{i + 1}} = 1}, \\ { - {}_{t_0}D_t^{{\alpha _{i + 1}}}{x_{i + 1}}\frac{{\partial f_i^{i + 1}}}{{\partial {x_{i + 1}}}}\prod \limits _{r = 1}^{i - 1} {{\rho _r},} }&{}{} \\ {{}_{t_0}D_t^{{\alpha _{i + 1}}}\left[ {{x_{i + 1}}g_i^{i}\prod \limits _{r = 1}^{i - 1} {{\rho _r}} } \right] }&{}{0< {\alpha _{i + 1}} < 1}. \\ { - {}_{t_0}D_t^{{\alpha _{i + 1}}}{x_{i + 1}}g_i^{i}\prod \limits _{r = 1}^{i - 1} {{\rho _r}}}&{} \\ {+ {}_{t_0}D_t^{{\alpha _{i + 1}}}f_i^i\prod \limits _{r = 1}^{i - 1} {{\rho _r}} ,}&{}{} \end{array}} \right. \end{aligned}$$

### Proof

To prove this theorem, we follow the $n-1$ steps mentioned in Theorem 1 to obtain ${}_{t_0}D_t^{{\alpha _n}| \cdots |{\alpha _{2}}|{\alpha _1}}$ as in the form of (25). Using the dynamics of the system to replace ${}_{t_0}D_t^{\alpha _1}$, we have

42 $$\begin{aligned}&{}_{t_0}D_t^{{\alpha _n}| \cdots |{\alpha _{2}}|{\alpha _1}}{x_1} \nonumber \\&\quad = {}_{{t_0}}D_t^{{\alpha _n}| \cdots |{\alpha _{2}}}\left[ {{}_{t_0}D_t^{{\alpha _1}}{x_1}} \right] \nonumber \\&\quad ={}_{t_0}D_t^{{\alpha _n}| \cdots |{\alpha _{2}}}\left\{ {\begin{array}{*{20}{l}} {f_1^{2} + {\varDelta _1}},&{}{{\alpha _{2}} {=} 1}, \\ {f_1^1 + g_1^{1}{x_{2}} + {\varDelta _1}},&{}{0 {<} {\alpha _{2}} {<} 1}. \end{array}} \right. \end{aligned}$$

By applying ${}_{t_0}D_t^{\alpha _{2}}$ and ${}_{t_0}D_t^{\alpha _{3}}$ to the expression in the single curly bracket in (42), as steps 1 and 2, one will obtain the relations in Boxes I and II, respectively. Examining (43) and (44) in Boxes I and II, respectively, one can discover the patterns based on which the terms develop till appearing in step $n-1$. These terms, in step *k*, include terms multiplied by ${}_{t_0}D_t^{\alpha _{k+1}}x_{k+1}$ which reveal the pattern for $\rho$, terms appeared in the curly brackets independent of $\varDelta _1,\varDelta _{2},\ldots ,\varDelta _{k+1}$ which reveal the pattern for $\mu$, and terms appeared in the curly brackets depending on $\varDelta _1,\varDelta _{2},\ldots ,\varDelta _{k+1}$ which reveal the pattern for $h_\varDelta ^{n-1}$ described in (30). Based on the discovered patterns from (43) and (44), one expects to obtain (29) in step $n-1$ where $\rho$ and $\mu$ will be in the forms described in (40) and (41), respectively, and $h_\varDelta ^{n-1}$ will take form as

**Table:** 

Box I	
$${}_{t_0}D_t^{{\alpha _{2}}|{\alpha _1}}{x_1}$$	
= $$\left\{ {\begin{array}{*{20}{l}} {{}_{t_0}D_t^{{\alpha _{2}}}f_1^{2} + \left\{ {{}_{t_0}D_t^{{\alpha _{2}}}{\varDelta _1}} \right\} ,}&{}{{\alpha _{2}} = 1}, \\ {{}_{t_0}D_t^{{\alpha _{2}}}\left[ {f_1^1 + g_1^{1}{x_{2}}} \right] + \left\{ {{}_{t_0}D_t^{{\alpha _{2}}}{\varDelta _1}} \right\} ,}&{}{0< {\alpha _{2}} < 1}, \end{array}} \right. $$	
= $$\left\{ {\begin{array}{*{20}{l}} {{}_{t_0}D_t^{{\alpha _{2}}}{x_{2}}\frac{{\partial f_1^{2}}}{{\partial {x_{2}}}} + \left\{ {{}_{t_0}D_t^{{\alpha _{2}}}f_1^{2} - {}_{t_0}D_t^{{\alpha _{2}}}{x_{2}}\frac{{\partial f_1^{2}}}{{\partial {x_{2}}}}} \right\} + \left\{ {{}_{t_0}D_t^{{\alpha _{2}}}{\varDelta _1}} \right\} ,}&{}{{\alpha _{2}} = 1}, \\ {{}_{t_0}D_t^{{\alpha _{2}}}{x_{2}}g_1^{1} + \left\{ {{}_{t_0}D_t^{{\alpha _{2}}}\left[ {{x_{2}}g_1^{1}} \right] - {}_{t_0}{D^{{\alpha _{2}}}}{x_{2}}g_1^{1}} \right\} + \left\{ {{}_{t_0}D_t^{{\alpha _{2}}}f_1^1} \right\} + \left\{ {{}_{t_0}D_t^{{\alpha _{2}}}{\varDelta _1}} \right\} ,}&{}{0< {\alpha _{2}} < 1}, \end{array}} \right. $$ (43)	
= $$\left\{ {\begin{array}{*{20}{l}} {\left[ {f_{2}^{3} + {\varDelta _{2}}} \right] \frac{{\partial f_1^{2}}}{{\partial {x_{2}}}} + \left\{ {{}_{t_0}D_t^{{\alpha _{2}}}f_1^{2} - {}_{t_0}D_t^{{\alpha _{2}}}{x_{2}}\frac{{\partial f_1^{2}}}{{\partial {x_{2}}}}} \right\} + \left\{ {{}_{t_0}D_t^{{\alpha _{2}}}{\varDelta _1}} \right\} ,}&{}{{\alpha _{2}} = 1,{\alpha _{3}} = 1}, \\ {\left[ {f_{2}^{2} + g_{2}^{2}{x_{3}} + {\varDelta _{2}}} \right] \frac{{\partial f_1^{2}}}{{\partial {x_{2}}}} + \left\{ {{}_{t_0}D_t^{{\alpha _{2}}}f_1^{2} - {}_{t_0}D_t^{{\alpha _{2}}}{x_{2}}\frac{{\partial f_1^{2}}}{{\partial {x_{2}}}}} \right\} + \left\{ {{}_{t_0}D_t^{{\alpha _{2}}}{\varDelta _1}} \right\} ,}&{}{{\alpha _{2}} = 1,0< {\alpha _{3}}< 1}, \\ {\left[ {f_{2}^{3} + {\varDelta _{2}}} \right] g_1^{1} + \left\{ {{}_{t_0}D_t^{{\alpha _{2}}}\left[ {{x_{2}}g_1^{1}} \right] - {}_{t_0}D_t^{{\alpha _{2}}}{x_{2}}g_1^{1}} \right\} + \left\{ {{}_{t_0}D_t^{{\alpha _{2}}}f_1^1} \right\} + \left\{ {{}_{t_0}D_t^{{\alpha _{2}}}{\varDelta _1}} \right\} ,}&{}{0< {\alpha _{2}}< 1,{\alpha _{3}} = 1}, \\ {\left[ {f_{2}^{2} + g_{2}^{2}{x_{3}} + {\varDelta _{2}}} \right] g_1^{1} + \left\{ {{}_{t_0}D_t^{{\alpha _{2}}}\left[ {{x_{2}}g_1^{1}} \right] - {}_{t_0}D_t^{{\alpha _{2}}}{x_{2}}g_1^{1}} \right\} + \left\{ {{}_{t_0}D_t^{{\alpha _{2}}}f_1^1} \right\} + \left\{ {{}_{t_0}D_t^{{\alpha _{2}}}{\varDelta _1}} \right\} ,}&{}{0< {\alpha _{2}}< 1,0< {\alpha _{3}} < 1}. \end{array}} \right. $$	

**Table:** 

Box II	
$$\begin{aligned}&{}^{}_{t_0}D_t^{{\alpha _{3}}|{\alpha _{2}}|{\alpha _1}}{x_1} \\&= \left\{ {\begin{array}{*{20}{l}} {{}^{}_{t_0}D_t^{{\alpha _{3}}}\left[ {f_{2}^{3}\frac{{\partial f_1^{2}}}{{\partial {x_{2}}}}} \right] + \left\{ {{}^{}_{t_0}D_t^{{\alpha _{3}}}\left[ {{\varDelta _{2}}\frac{{\partial f_1^{2}}}{{\partial {x_{2}}}}} \right] } \right\} }&{}{{\alpha _{2}} = 1,{\alpha _{3}} = 1}, \\ { + \left\{ {{}^{}_{t_0}D_t^{{\alpha _{3}}}\left[ {{}^{}_{t_0}D_t^{{\alpha _{2}}}f_1^{2} - {}^{}_{t_0}D_t^{{\alpha _{2}}}{x_{2}}\frac{{\partial f_1^{2}}}{{\partial {x_{2}}}}} \right] } \right\} + \left\{ {{}^{}_{t_0}D_t^{{\alpha _{2}} + {\alpha _{3}}}{\varDelta _1}} \right\} ,}&{}{} \\ {{}^{}_{t_0}D_t^{{\alpha _{3}}}\left[ {{x_{3}}g_{2}^{2}\frac{{\partial f_1^{2}}}{{\partial {x_{2}}}}} \right] + \left\{ {{}^{}_{t_0}D_t^{{\alpha _{3}}}\left[ {f_{2}^{2}\frac{{\partial f_1^{2}}}{{\partial {x_{2}}}}} \right] } \right\} + \left\{ {{}^{}_{t_0}D_t^{{\alpha _{3}}}\left[ {{\varDelta _{2}}\frac{{\partial f_1^{2}}}{{\partial {x_{2}}}}} \right] } \right\} }&{}{{\alpha _{2}} = 1,0< {\alpha _{3}}< 1}, \\ { + \left\{ {{}^{}_{t_0}D_t^{{\alpha _{3}}}\left[ {{}^{}_{t_0}D_t^{{\alpha _{2}}}f_1^{2} - {}^{}_{t_0}D_t^{{\alpha _{2}}}{x_{2}}\frac{{\partial f_1^{2}}}{{\partial {x_{2}}}}} \right] } \right\} + \left\{ {{}^{}_{t_0}D_t^{{\alpha _{2}} + {\alpha _{3}}}{\varDelta _1}} \right\} ,}&{}{} \\ {{}^{}_{t_0}D_t^{{\alpha _{3}}}\left[ {f_{2}^{3}g_1^{1}} \right] + \left\{ {{}^{}_{t_0}D_t^{{\alpha _{3}}}\left[ {{\varDelta _{2}}g_1^{1 }} \right] } \right\} }&{}{0< {\alpha _{2}}< 1,{\alpha _{3}} = 1}, \\ { + \left\{ {{}^{}_{t_0}D_t^{{\alpha _{3}}}\left[ {{}^{}_{t_0}D_t^{{\alpha _{2}}}\left[ {{x_{2}}g_1^{1}} \right] - {}^{}_{t_0}D_t^{{\alpha _{2}}}{x_{2}}g_1^{1}} \right] } \right\} + \left\{ {{}^{}_{t_0}D_t^{{\alpha _{2}} + {\alpha _{3}}}f_1^1} \right\} + \left\{ {{}^{}_{t_0}D_t^{{\alpha _{2}} + {\alpha _{3}}}{\varDelta _1}} \right\} ,}&{}{} \\ {{}^{}_{t_0}D_t^{{\alpha _{3}}}\left[ {{x_{3}}g_{2}^{2}g_1^{1}} \right] + \left\{ {{}^{}_{t_0}D_t^{{\alpha _{3}}}\left[ {f_{2}^{2}g_1^{1 }} \right] } \right\} + \left\{ {{}^{}_{t_0}D_t^{{\alpha _{3}}}\left[ {{\varDelta _{2}}g_1^{1}} \right] } \right\} }&{}{0< {\alpha _{2}}< 1,0< {\alpha _{3}}< 1}, \\ { + \left\{ {{}^{}_{t_0}D_t^{{\alpha _{3}}}\left[ {{}^{}_{t_0}D_t^{{\alpha _{2}}}\left[ {{x_{2}}g_1^{1}} \right] - {}^{}_{t_0}D_t^{{\alpha _{2}}}{x_{2}}g_1^{1}} \right] } \right\} + \left\{ {{}^{}_{t_0}D_t^{{\alpha _{2}} + {\alpha _{3}}}f_1^1} \right\} + \left\{ {{}^{}_{t_0}D_t^{{\alpha _{2}} + {\alpha _{3}}}{\varDelta _1}} \right\} ,}&{}{} \end{array}} \right. \\&= \left\{ {\begin{array}{*{20}{l}} {{}^{}_{t_0}D_t^{{\alpha _{3}}}{x_{3}}\frac{{\partial f_{2}^{3}}}{{\partial {x_{3}}}}\frac{{\partial f_1^{2}}}{{\partial {x_{2}}}} + \left\{ {{}^{}_{t_0}D_t^{{\alpha _{3}}}\left[ {f_{2}^{3}\frac{{\partial f_1^{2}}}{{\partial {x_{2}}}}} \right] - {}^{}_{t_0}D_t^{{\alpha _{3}}}{x_{3}}\frac{{\partial f_{2}^{3}}}{{\partial {x_{3}}}}\frac{{\partial f_1^{2}}}{{\partial {x_{2}}}}} \right\} }&{}{{\alpha _{2}} = 1,{\alpha _{3}} = 1}, \\ { + \left\{ {{}^{}_{t_0}D_t^{{\alpha _{3}}}\left[ {{\varDelta _{2}}\frac{{\partial f_1^{2}}}{{\partial {x_{2}}}}} \right] } \right\} + \left\{ {{}^{}_{t_0}D_t^{{\alpha _{3}}}\left[ {{}^{}_{t_0}D_t^{{\alpha _{2}}}f_1^{2} - {}^{}_{t_0}D_t^{{\alpha _{2}}}{x_{2}}\frac{{\partial f_1^{2}}}{{\partial {x_{2}}}}} \right] } \right\} + \left\{ {{}^{}_{t_0}D_t^{{\alpha _{2}} + {\alpha _{3}}}{\varDelta _1}} \right\} ,}&{}{} \\ {{}^{}_{t_0}D_t^{{\alpha _{3}}}{x_{3}}g_{2}^{2}\frac{{\partial f_1^{2}}}{{\partial {x_{2}}}} + \left\{ {{}^{}_{t_0}D_t^{{\alpha _{3}}}\left[ {{x_{3}}g_{2}^{2}\frac{{\partial f_1^{2}}}{{\partial {x_{2}}}}} \right] - {}^{}_{t_0}D_t^{{\alpha _{3}}}{x_{3}}g_{2}^{2}\frac{{\partial f_1^{2}}}{{\partial {x_{2}}}}} \right\} + \left\{ {{}^{}_{t_0}D_t^{{\alpha _{3}}}\left[ {f_{2}^{2}\frac{{\partial f_1^{2}}}{{\partial {x_{2}}}}} \right] } \right\} }&{}{{\alpha _{2}} = 1,0< {\alpha _{3}}< 1}, \\ { + \left\{ {{}^{}_{t_0}D_t^{{\alpha _{3}}}\left[ {{\varDelta _{2}}\frac{{\partial f_1^{2}}}{{\partial {x_{2}}}}} \right] } \right\} + \left\{ {{}^{}_{t_0}D_t^{{\alpha _{3}}}\left[ {{}^{}_{t_0}D_t^{{\alpha _{2}}}f_1^{2} - {}^{}_{t_0}D_t^{{\alpha _{2}}}{x_{2}}\frac{{\partial f_1^{2}}}{{\partial {x_{2}}}}} \right] } \right\} + \left\{ {{}^{}_{t_0}D_t^{{\alpha _{2}} + {\alpha _{3}}}{\varDelta _1}} \right\} ,}&{}{} \\ {{}^{}_{t_0}D_t^{{\alpha _{3}}}{x_{3}}\frac{{\partial f_{2}^{3}}}{{\partial {x_{3}}}}g_1^{1} + \left\{ {{}^{}_{t_0}D_t^{{\alpha _{3}}}\left[ {f_{2}^{3}g_1^{1}} \right] - {}^{}_{t_0}D_t^{{\alpha _{3}}}{x_{3}}\frac{{\partial f_{2}^{3}}}{{\partial {x_{3}}}}g_1^{1}} \right\} + \left\{ {{}^{}_{t_0}D_t^{{\alpha _{3}}}\left[ {{\varDelta _{2}}g_1^{1}} \right] } \right\} }&{}{0< {\alpha _{2}}< 1,{\alpha _{3}} = 1}, \\ { + \left\{ {{}^{}_{t_0}D_t^{{\alpha _{3}}}\left[ {{}^{}_{t_0}D_t^{{\alpha _{2}}}\left[ {{x_{2}}g_1^{1}} \right] - {}^{}_{t_0}D_t^{{\alpha _{2}}}{x_{2}}g_1^{1}} \right] } \right\} + \left\{ {{}^{}_{t_0}D_t^{{\alpha _{2}} + {\alpha _{3}}}f_1^1} \right\} + \left\{ {{}^{}_{t_0}D_t^{{\alpha _{2}} + {\alpha _{3}}}{\varDelta _1}} \right\} ,}&{}{} \\ {{}^{}_{t_0}D_t^{{\alpha _{3}}}{x_{3}}g_{2}^{2}g_1^{1} + \left\{ {{}^{}_{t_0}D_t^{{\alpha _{3}}}\left[ {{x_{3}}g_{2}^{2}g_1^{1}} \right] - {}^{}_{t_0}D_t^{{\alpha _{3}}}{x_{3}}g_{2}^{2}g_1^{1}} \right\} + \left\{ {{}^{}_{t_0}D_t^{{\alpha _{3}}}\left[ {f_{2}^{2}g_1^{1}} \right] } \right\} }&{}{0< {\alpha _{2}}< 1,0< {\alpha _{3}}< 1}, \\ { + \left\{ {{}^{}_{t_0}D_t^{{\alpha _{3}}}\left[ {{\varDelta _{2}}g_1^{1}} \right] } \right\} + \left\{ {{}^{}_{t_0}D_t^{{\alpha _{3}}}\left[ {{}^{}_{t_0}D_t^{{\alpha _{2}}}\left[ {{x_{2}}g_1^{1}} \right] - {}^{}_{t_0}D_t^{{\alpha _{2}}}{x_{2}}g_1^{1}} \right] } \right\} + \left\{ {{}^{}_{t_0}D_t^{{\alpha _{2}} + {\alpha _{3}}}f_1^1} \right\} + \left\{ {{}^{}_{t_0}D_t^{{\alpha _{2}} + {\alpha _{3}}}{\varDelta _1}} \right\} ,}&{}{} \end{array}} \right. \\&= \left\{ {\begin{array}{*{20}{l}} {\left[ {f_{3}^{4} + {\varDelta _{3}}} \right] \frac{{\partial f_{2}^{3}}}{{\partial {x_{3}}}}\frac{{\partial f_1^{2}}}{{\partial {x_{2}}}} + \left\{ {{}^{}_{t_0}D_t^{{\alpha _{3}}}\left[ {f_{2}^{3}\frac{{\partial f_1^{2}}}{{\partial {x_{2}}}}} \right] - {}^{}_{t_0}D_t^{{\alpha _{3}}}{x_{3}}\frac{{\partial f_{2}^{3}}}{{\partial {x_{3}}}}\frac{{\partial f_1^{2}}}{{\partial {x_{2}}}}} \right\} }&{}{{\alpha _{2}} = 1,{\alpha _{3}} = 1,{\alpha _{4}} = 1}, \\ { + \left\{ {{}^{}_{t_0}D_t^{{\alpha _{3}}}\left[ {{\varDelta _{2}}\frac{{\partial f_1^{2}}}{{\partial {x_{2}}}}} \right] } \right\} + \left\{ {{}^{}_{t_0}D_t^{{\alpha _{3}}}\left[ {{}^{}_{t_0}D_t^{{\alpha _{2}}}f_1^{2} - {}^{}_{t_0}D_t^{{\alpha _{2}}}{x_{2}}\frac{{\partial f_1^{2}}}{{\partial {x_{2}}}}} \right] } \right\} + \left\{ {{}^{}_{t_0}D_t^{{\alpha _{2}} + {\alpha _{3}}}{\varDelta _1}} \right\} ,}&{}{} \\ {\left[ {f_{3}^{3} + g_{3}^{3}{x_{4}} + {\varDelta _{3}}} \right] \frac{{\partial f_{2}^{3}}}{{\partial {x_{3}}}}\frac{{\partial f_1^{2}}}{{\partial {x_{2}}}} + \left\{ {{}^{}_{t_0}D_t^{{\alpha _{3}}}\left[ {f_{2}^{3}\frac{{\partial f_1^{2}}}{{\partial {x_{2}}}}} \right] - {}^{}_{t_0}D_t^{{\alpha _{3}}}{x_{3}}\frac{{\partial f_{2}^{3}}}{{\partial {x_{3}}}}\frac{{\partial f_1^{2}}}{{\partial {x_{2}}}}} \right\} }&{}{{\alpha _{2}} = 1,{\alpha _{3}} = 1,0< {\alpha _{4}}< 1}, \\ { + \left\{ {{}^{}_{t_0}D_t^{{\alpha _{3}}}\left[ {{\varDelta _{2}}\frac{{\partial f_1^{2}}}{{\partial {x_{2}}}}} \right] } \right\} + \left\{ {{}^{}_{t_0}D_t^{{\alpha _{3}}}\left[ {{}^{}_{t_0}D_t^{{\alpha _{2}}}f_1^{2} - {}^{}_{t_0}D_t^{{\alpha _{2}}}{x_{2}}\frac{{\partial f_1^{2}}}{{\partial {x_{2}}}}} \right] } \right\} + \left\{ {{}^{}_{t_0}D_t^{{\alpha _{2}} + {\alpha _{3}}}{\varDelta _1}} \right\} ,}&{}{} \\ {\left[ {f_{3}^{4} + {\varDelta _{3}}} \right] g_{2}^{2}\frac{{\partial f_1^{2}}}{{\partial {x_{2}}}} + \left\{ {{}^{}_{t_0}D_t^{{\alpha _{3}}}\left[ {{x_{3}}g_{2}^{2}\frac{{\partial f_1^{2}}}{{\partial {x_{2}}}}} \right] - {}^{}_{t_0}D_t^{{\alpha _{3}}}{x_{3}}g_{2}^{2}\frac{{\partial f_1^{2}}}{{\partial {x_{2}}}}} \right\} + \left\{ {{}^{}_{t_0}D_t^{{\alpha _{3}}}\left[ {f_{2}^{2}\frac{{\partial f_1^{2}}}{{\partial {x_{2}}}}} \right] } \right\} }&{}{{\alpha _{2}} = 1,0< {\alpha _{3}}< 1,{\alpha _{4}} = 1}, \\ { + \left\{ {{}^{}_{t_0}D_t^{{\alpha _{3}}}\left[ {{\varDelta _{2}}\frac{{\partial f_1^{2}}}{{\partial {x_{2}}}}} \right] } \right\} + \left\{ {{}^{}_{t_0}D_t^{{\alpha _{3}}}\left[ {{}^{}_{t_0}D_t^{{\alpha _{2}}}f_1^{2} - {}^{}_{t_0}D_t^{{\alpha _{2}}}{x_{2}}\frac{{\partial f_1^{2}}}{{\partial {x_{2}}}}} \right] } \right\} + \left\{ {{}^{}_{t_0}D_t^{{\alpha _{2}} + {\alpha _{3}}}{\varDelta _1}} \right\} ,}&{}{} \\ {\left[ {f_{3}^{3} + g_{3}^{3}{x_{4}} + {\varDelta _{3}}} \right] g_{2}^{2}\frac{{\partial f_1^{2}}}{{\partial {x_{2}}}} + \left\{ {{}^{}_{t_0}D_t^{{\alpha _{3}}}\left[ {{x_{3}}g_{2}^{2}\frac{{\partial f_1^{2}}}{{\partial {x_{2}}}}} \right] - {}^{}_{t_0}D_t^{{\alpha _{3}}}{x_{3}}g_{2}^{2}\frac{{\partial f_1^{2}}}{{\partial {x_{2}}}}} \right\} + \left\{ {{}^{}_{t_0}D_t^{{\alpha _{3}}}\left[ {f_{2}^{2}\frac{{\partial f_1^{2}}}{{\partial {x_{2}}}}} \right] } \right\} }&{}{{\alpha _{2}} = 1,0< {\alpha _{3}}< 1,0< {\alpha _{4}}< 1}, \\ { + \left\{ {{}^{}_{t_0}D_t^{{\alpha _{3}}}\left[ {{\varDelta _{2}}\frac{{\partial f_1^{2}}}{{\partial {x_{2}}}}} \right] } \right\} + \left\{ {{}^{}_{t_0}D_t^{{\alpha _{3}}}\left[ {{}^{}_{t_0}D_t^{{\alpha _{2}}}f_1^{2} - {}^{}_{t_0}D_t^{{\alpha _{2}}}{x_{2}}\frac{{\partial f_1^{2}}}{{\partial {x_{2}}}}} \right] } \right\} + \left\{ {{}^{}_{t_0}D_t^{{\alpha _{2}} + {\alpha _{3}}}{\varDelta _1}} \right\} ,}&{}{} \\ {\left[ {f_{3}^{4} + {\varDelta _{3}}} \right] \frac{{\partial f_{2}^{3}}}{{\partial {x_{3}}}}g_1^{1} + \left\{ {{}^{}_{t_0}D_t^{{\alpha _{3}}}\left[ {f_{2}^{3}g_1^{1}} \right] - {}^{}_{t_0}D_t^{{\alpha _{3}}}{x_{3}}\frac{{\partial f_{2}^{3}}}{{\partial {x_{3}}}}g_1^{1}} \right\} + \left\{ {{}^{}_{t_0}D_t^{{\alpha _{3}}}\left[ {{\varDelta _{2}}g_1^{1}} \right] } \right\} }&{}{0< {\alpha _{2}}< 1,{\alpha _{3}} = 1,{\alpha _{4}} = 1}, \\ { + \left\{ {{}^{}_{t_0}D_t^{{\alpha _{3}}}\left[ {{}^{}_{t_0}D_t^{{\alpha _{2}}}\left[ {{x_{2}}g_1^{1}} \right] - {}^{}_{t_0}D_t^{{\alpha _{2}}}{x_{2}}g_1^{1}} \right] } \right\} + \left\{ {{}^{}_{t_0}D_t^{{\alpha _{2}} + {\alpha _{3}}}f_1^1} \right\} + \left\{ {{}^{}_{t_0}D_t^{{\alpha _{2}} + {\alpha _{3}}}{\varDelta _1}} \right\} ,}&{}{} \\ {\left[ {f_{3}^{3} + g_{3}^{3}{x_{4}} + {\varDelta _{3}}} \right] \frac{{\partial f_{2}^{3}}}{{\partial {x_{3}}}}g_1^{1} + \left\{ {{}^{}_{t_0}D_t^{{\alpha _{3}}}\left[ {f_{2}^{3}g_1^{1}} \right] - {}^{}_{t_0}D_t^{{\alpha _{3}}}{x_{3}}\frac{{\partial f_{2}^{3}}}{{\partial {x_{3}}}}g_1^{1}} \right\} + \left\{ {{}^{}_{t_0}D_t^{{\alpha _{3}}}\left[ {{\varDelta _{2}}g_1^{1}} \right] } \right\} }&{}{0< {\alpha _{2}}< 1,{\alpha _{3}} = 1,0< {\alpha _{4}}< 1}, \\ { + \left\{ {{}^{}_{t_0}D_t^{{\alpha _{3}}}\left[ {{}^{}_{t_0}D_t^{{\alpha _{2}}}\left[ {{x_{2}}g_1^{1}} \right] - {}^{}_{t_0}D_t^{{\alpha _{2}}}{x_{2}}g_1^{1}} \right] } \right\} + \left\{ {{}^{}_{t_0}D_t^{{\alpha _{2}} + {\alpha _{3}}}f_1^1} \right\} + \left\{ {{}^{}_{t_0}D_t^{{\alpha _{2}} + {\alpha _{3}}}{\varDelta _1}} \right\} ,}&{}{} \\ {\left[ {f_{3}^{4} + {\varDelta _{3}}} \right] g_{2}^{2}g_1^{1} + \left\{ {{}^{}_{t_0}D_t^{{\alpha _{3}}}\left[ {{x_{3}}g_{2}^{2}g_1^{1}} \right] - {}^{}_{t_0}D_t^{{\alpha _{3}}}{x_{3}}g_{2}^{2}g_1^{1}} \right\} + \left\{ {{}^{}_{t_0}D_t^{{\alpha _{3}}}\left[ {f_{2}^{2}g_1^{1}} \right] } \right\} }&{}{0< {\alpha _{2}}< 1,0< {\alpha _{3}}< 1,{\alpha _{4}} = 1}, \\ { + \left\{ {{}^{}_{t_0}D_t^{{\alpha _{3}}}\left[ {{\varDelta _{2}}g_1^{1}} \right] } \right\} + \left\{ {{}^{}_{t_0}D_t^{{\alpha _{3}}}\left[ {{}^{}_{t_0}D_t^{{\alpha _{2}}}\left[ {{x_{2}}g_1^{1}} \right] - {}^{}_{t_0}D_t^{{\alpha _{2}}}{x_{2}}g_1^{1}} \right] } \right\} + \left\{ {{}^{}_{t_0}D_t^{{\alpha _{2}} + {\alpha _{3}}}f_1^1} \right\} + \left\{ {{}^{}_{t_0}D_t^{{\alpha _{2}} + {\alpha _{3}}}{\varDelta _1}} \right\} ,}&{}{} \\ {\left[ {f_{3}^{3} + g_{3}^{3}{x_{4}} + {\varDelta _{3}}} \right] g_{2}^{2}g_1^{1} + \left\{ {{}^{}_{t_0}D_t^{{\alpha _{3}}}\left[ {{x_{3}}g_{2}^{2}g_1^{1 }} \right] - {}^{}_{t_0}D_t^{{\alpha _{3}}}{x_{3}}g_{2}^{2}g_1^{1}} \right\} + \left\{ {{}^{}_{t_0}D_t^{{\alpha _{3}}}\left[ {f_{2}^{2}g_1^{1}} \right] } \right\} }&{}{0< {\alpha _{2}}< 1,0< {\alpha _{3}}< 1,0< {\alpha _{4}} < 1}. \\ { + \left\{ {{}^{}_{t_0}D_t^{{\alpha _{3}}}\left[ {{\varDelta _{2}}g_1^{1}} \right] } \right\} + \left\{ {{}^{}_{t_0}D_t^{{\alpha _{3}}}\left[ {{}^{}_{t_0}D_t^{{\alpha _{2}}}\left[ {{x_{2}}g_1^{1}} \right] - {}^{}_{t_0}D_t^{{\alpha _{2}}}{x_{2}}g_1^{1}} \right] } \right\} + \left\{ {{}^{}_{t_0}D_t^{{\alpha _{2}} + {\alpha _{3}}}f_1^1} \right\} + \left\{ {{}^{}_{t_0}D_t^{{\alpha _{2}} + {\alpha _{3}}}{\varDelta _1}} \right\} ,}&{}{} \end{array}} \right. \end{aligned}$$	(44)

45 $$\begin{aligned} h_\varDelta ^{n - 1} = \sum \limits _{k = 1}^{n - 1} {{}_{t_0}D_t^{{\alpha _n}| \cdots |{\alpha _{k + 1}}}\left[ {{\varDelta _k}\prod \limits _{r = 1}^{k - 1} {{\rho _r}} } \right] } . \end{aligned}$$

By replacing (45) and ${}_{t_0}D_t^{\alpha _{n}}x_{n}$, using the dynamics of the system, in (29) one gets (25) where $h_\varDelta$ is obtained with (6). $\square$

The following remark gives some hints to select the design parameters of the offered controller.

### Remark 2

In Theorem 1, as for $\gamma$ and $\beta$, choosing $\gamma =1$ and non-negative integers for $\beta$ which lead to integer-order operators reduces computational burden compared to other values which lead to FO operators. Moreover, it is obvious that there are infinite choices for the values of $\beta$ and *m* for which (16) and (17) hold. It is suggested that the values for $\beta$ and *m* be selected in such a way that $\beta + \sum \nolimits _{k = 1}^n {{\alpha _k}} \approx m$ holds. This selection causes the FO polynomial in (35), from which (18) is originated, to tend to an integer-order polynomial whose dynamical behavior tuning is more convenient via selecting appropriate values for $c_l$ to place its roots at desired points. In the case of $\alpha _1=\alpha _{2}=\cdots =\alpha _n=1$, the selections of $\beta =0$ and $m=n$ are the most convenient. It follows that there is no need for *m* to be selected larger than *n*.

With regard to Theorems 1 and 2 and Remark 2, the following algorithm is suggested to obtain the parameters and the SMC law for the system in (4).

**Design Algorithm:**

1. Obtain $\rho$, $\mu$, and $h_\varDelta$ according to (40), (41), and (6).
2. Choose values for $\gamma \in (0,1]$, preferably $\gamma =1$, and $k_s\in (0,\infty )$.
3. If $\alpha _1=\alpha _{2}=\cdots =\alpha _n=1$, select $\beta =0$ and $m=n$, and go to 5. Otherwise, go to the next step.
4. Choose values for $\beta \ge 0$, preferably an integer, and $m\in \{1,2,\ldots ,n\}$ such that (17) holds while $\beta + \sum \nolimits _{k = 1}^n {{\alpha _k}}$ is close to *m* as much as possible.
5. Calculate $c_0, c_1,\ldots ,c_{m-1}$ such that the roots of (18) satisfy (19). 6. Obtain *S*(*t*) and *u* according to (15) and (20).

The SMC design method presented in this work is based on designing one sliding surface in one step, based on the presented closed-form solution, rather than designing sliding surfaces and virtual inputs in *n* steps. This increases the simplicity and applicability of the method to large extend. The following remark reveals these advantages compared to the state-of-the-art.

### Remark 3

A comparison between the SMC design method suggested in this work for the FO nonstrict-feedback nonlinear systems formed as (4) and the only SMC design method in the literature offered for these systems by the work of [31], as the state-of-the-art, is presented as follows:

- The number of design parameters in this work, in a worst-case scenario, is $n+4$ ($k_s$, $\gamma$, *m*, $\beta$, and $c_l$ where $l=0,1,\ldots ,n-1$), but that in [31], excluding the design parameters used in the both fuzzy system and adaptive laws, is $3n+4$ ($c_i$, $k_{1i}$, $k_{2i}$, *k*, $\xi$, *r*, and *q* where $i=1,2,\ldots ,n$).
- In this work, since $0<\alpha _i\le 1$ holds for $i=1,2,\ldots ,n$, it is applicable to the systems of both commensurate and incommensurate orders and also systems with a mixture of integer-order and FO dynamics. However, in [31], since $\alpha _i=\alpha$ for $i=1,2,\ldots ,n$ and $0<\alpha < 1$ hold, it is applicable only to the systems of commensurate order and systems with only FO dynamics.
- In this work, $0<\alpha _i\le 1$ holds for $i=1,2,\ldots ,n$. Therefore, it is applicable to the systems with derivatives of any order. However, the work of [31] is applicable to the systems with $0<\alpha <1$ and $3/4<\alpha (1+\alpha )$, that is, only the systems with $0.83\le \alpha <1$.

Though this article focused merely on a new SMC design method for FO nonstrict-feedback nonlinear systems, issues such as saturation, fault, and estimation of disturbances and uncertainties could be incorporated into the presented design method with different techniques introduced in the literature [26, 31, 36], which could be further investigated by interested researchers.

## Numerical example

In this section, the efficiency of the control law design method offered in the last section is illustrated by applying it to a model arising from a practical loudspeaker. In [24], the FO model of a loudspeaker was identified as

46 $$\begin{aligned}&u\!\left( t \right) = {R_e}i\!\left( t \right) + Bl\!\left( x \right) \frac{{dx\!\left( t \right) }}{{dt}} + {}{_0}D_t^{b }\left[ {{L_\beta }\!\left( x \right) i\!\left( t \right) } \right] , \end{aligned}$$

47 $$\begin{aligned}&Bl\!\left( x \right) i\!\left( t \right) = {M_t}\frac{{{d^2}x\!\left( t \right) }}{{d{t^2}}} + {R_m}\frac{{dx\!\left( t \right) }}{{dt}} + \eta \!\left( x \right) {}{_0}D_t^a x\!\left( t \right) \nonumber \\&\qquad \qquad \qquad + K\!\left( x \right) x\!\left( t \right) - \frac{{{i^2}\!\left( t \right) }}{2}\frac{{d{L_\beta }\!\left( x \right) }}{{dx}}, \end{aligned}$$

48 $$\begin{aligned}&y\!\left( t\right) = x\!\left( t\right) , \end{aligned}$$

where the input voltage, *u*(*t*), and the cone displacement, *x*(*t*), are the input and the output, respectively. The superiority of this model over conventional integer-order models was shown by the aforementioned work both numerically and experimentally. As can be seen, this model is a mixture of integer-order and FO derivatives. While there is no SMC design method in the literature for such a model, in the following, a SMC law is designed for this model using the theorems presented in the last section. One can check that the model in (46)–(48), after some manipulation, can be formulated as (4) where

$$\begin{aligned}&n = 4,{\alpha _1} = a,{\alpha _2} = 1 - a,{\alpha _3} = 1,\\&{\alpha _4} = b,{c_y} = 1,j=\varnothing , \end{aligned}$$

$$\begin{aligned}&{x_1} = x\!\left( t \right) ,{x_2} = {}{_0}D_t^a x\!\left( t \right) , {x_3} = dx\!\left( t \right) /dt,\\&{x_4} = {L_\beta }\!\left( x \right) i\!\left( t \right) , \end{aligned}$$

$$\begin{aligned} f_1^1 = 0,g_1^1 = 1,\varDelta _1=0,f_2^3 = {x_3},\varDelta _2=0, \end{aligned}$$

$$\begin{aligned} f_3^3 = - \frac{{K\!\left( {{x_1}} \right) }}{{{M_t}}}{x_1} - \frac{{\eta \!\left( {{x_1}} \right) }}{{{M_t}}}{x_2} - \frac{{{R_m}}}{{{M_t}}}{x_3}, \end{aligned}$$

$$\begin{aligned} g_3^3 = \frac{{Bl\!\left( {{x_1}} \right) }}{{{M_t}{L_\beta }\!\left( {{x_1}} \right) }}, \varDelta _3= \frac{{{dL_\beta }\!\left( {{x_1}} \right) }/dx_1}{{2{M_t}L_\beta ^2\!\left( {{x_1}} \right) }}{x^2_4}, \end{aligned}$$

$$\begin{aligned} {f_4} = - Bl\!\left( {{x_1}} \right) {x_3} - \frac{{{R_e}}}{{{L_\beta }\!\left( {{x_1}} \right) }}{x_4},{g_4} = 1, \end{aligned}$$

whose the numerical values of the relevant parameters are

$$\begin{aligned} a = 0.116,b = 0.890, \end{aligned}$$

$$\begin{aligned} {R_e} = 7.23,{M_t} = 0.582 \times {10^{ - 3}},{R_m} = 0.089, \end{aligned}$$

$$\begin{aligned} Bl\left( {{x_1}} \right) =&- 0.4163x_1^4 - 0.2567x_1^3 - 0.3172x_1^2 \\&+ 0.0295{x_1}+ 2.5479, \end{aligned}$$

$$\begin{aligned} {L_\beta }\left( {{x_1}} \right) = {10^{ - 3}}&( - 0.0636x_1^4 - 0.0113x_1^3 \\&- 0.0305x_1^2 - 0.0735{x_1} + 0.5824 ), \end{aligned}$$

$$\begin{aligned} K\left( {{x_1}} \right) = {10^2}&( 0.3561x_1^4 - 0.1619x_1^3 \\&+ 0.6245x_1^2 + 0.4177{x_1}+ 1.9999 ), \end{aligned}$$

$$\begin{aligned} \eta \left( {{x_1}} \right) = {10^2}&( - 0.8325x_1^4 + 0.1665x_1^3 + 1.3070x_1^2 \\&+ 2.2561{x_1}+ 4.0854 ). \end{aligned}$$

Note that $f_3^3$, $g_3^3$, and $\varDelta _3$ have been determined using Remark 1. For a conventional loudspeaker, the parameters of $Bl(x_1)$, $L_\beta (x_1)$, $K(x_1)$, and $\eta (x_1)$ are positive, and their derivatives exit and are bounded [14]. Therefore, $f_1^1$, $g_1^1$, $f_2^3$, $f_3^3$, $g_3^3$, $f_4$, and $g_4$ are continuous and locally Lipschitz. Considering that $| x_1| \le 0.5$ mm holds in practice, this system meets Assumption 1. Although no uncertainties was considered by [24] in the fourth equation, assume

$$\begin{aligned} {\varDelta _4} = {x_1}\cos \left( t \right) + 0.2\sin \left( {200\pi t} \right) . \end{aligned}$$

Using Theorem 2, we have

$$\begin{aligned} {\rho _1} = {\rho _2} = 1,{\rho _3} = \rho = g_3^3, \end{aligned}$$

$$\begin{aligned} {\mu _1}&= {\mu _2} = 0, {\mu _3} = \mu = {}{_0}D_t^{\alpha _4}\left[ {{x_4}g_3^3} \right] \\&\quad - {}{_0}D_t^{\alpha _4} {x_4} g_3^3 + {}{_0}D_t^{\alpha _4} f_3^3, \end{aligned}$$

$$\begin{aligned} {h_\varDelta } = {}{_0}D_t^{\alpha _4} \varDelta _3+\varDelta _4 \rho _3. \end{aligned}$$

**Fig. 1:**
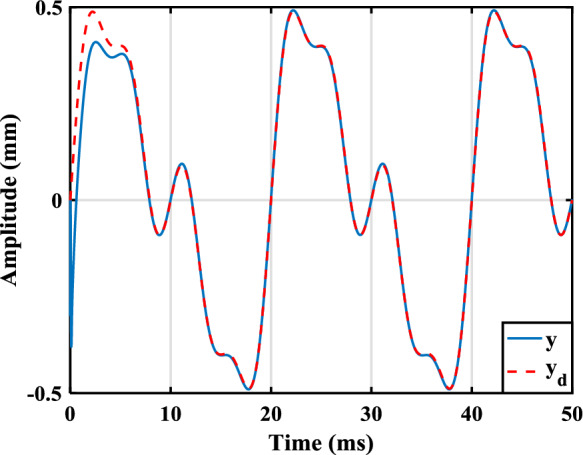
The output (blue solid line) and reference input (red dashed line) of (46)–(48), in millimeter, using the SMC law designed based on Theorems 1 and 2

**Fig. 2:**
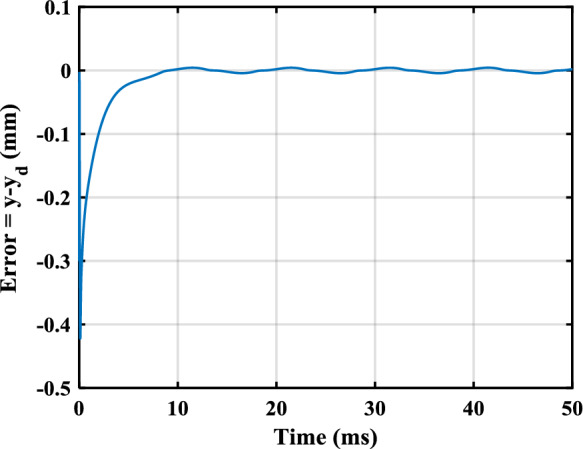
Output tracking error

**Fig. 3:**
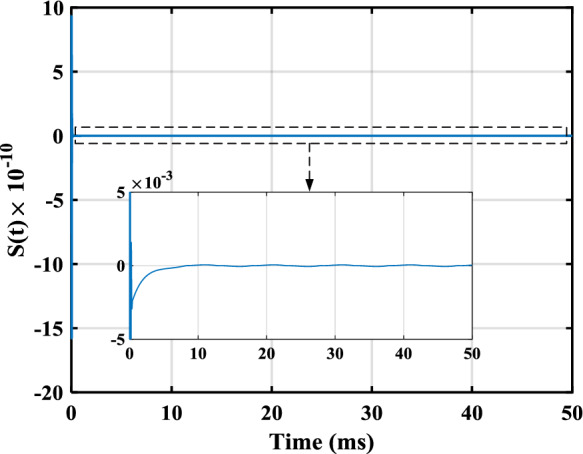
Sliding surface, *S*(*t*)

Regarding Remark 2 and Theorem 1, let $\gamma =1$, $\beta =0$, and $m=3$. Consider also $k_s=500$. Moreover, to place the roots of (18) at $-5\times 10^3$, one has $c_0=1.25\times 10^8$, $c_1=7.5\times 10^7$, and $c_2=1.5\times 10^4$. Further, let $c_\varDelta =4.7\times 10^8$ to meet Assumption 2. Suppose $y_d=10^{-3}[{0.4\sin ( {100\pi t})}+{0.2\sin ( {200\pi t})}+{0.1\sin ( {400\pi t})}]$ which satisfies Assumption 3. The calculated parameters are substituted into the control law described in (20)–(22), and the control law is applied to (46)–(48). The simulations are performed using the Simulink of MATLAB, and the FO derivative operators are implemented using Toolkit [21]. The sampling time rate and initial values are chosen as 96 kHz (a standard rate in audio applications) and $[-3\times 10^{-3},0,0,0]$, respectively. The output is obtained as displayed in Fig. 1, where the blue solid line and the red dashed line show the output and the reference input, respectively. As can be seen, the output asymptotically tracks the reference input. The tracking error and the sliding surface have been also plotted in Figs. 2 and 3.

According to Remark 3, while the SMC design method suggested here was applied to the system in (46)–(48) easily, the one presented by [31] is not applicable to this system because of three reasons: $\alpha _1\ne \alpha _2\ne \alpha _3\ne \alpha _4$; $\alpha _4<0.83$; and $\alpha _3=1$. Moreover, the number of design parameters in the method offered here for this example is 7, but that in the method introduced by [31] for a system with $n=4$ is 16.

## Conclusion

Unlike the Leibniz rule for integer-order derivatives of the product of two functions which includes only two terms, the rule for FO derivatives of that includes an infinite number of terms. This challenge caused the sliding mode control (SMC) design methods introduced in the literature so far to be applicable to a very limited class of FO nonlinear systems. In this article, it was shown that only one of these infinite terms is needed to design a SMC law for a class of incommensurate FO nonstrict-feedback systems, and thereby, an algorithm was offered to design a new SMC design method which decreased the number of design parameters and increased the applicability of the method to large extend, compared to the state-of-the-are. Stability and finite-time convergence of the suggested method was proved. Moreover, a closed-form solution was presented for the algorithm which offers the designer a simple tool to design the controller. The merit of the presented design method was illustrated through a numerical example.

## Data Availability

Data sharing not applicable to this article as no data sets were generated or analyzed during the current study.
